# Bioprostheses and Mechanical Prostheses for Aortic Valve Replacement in Patients Aged 50 to 65 Years Offer Similar Long-Term Survival Rates

**DOI:** 10.3390/jcdd12020044

**Published:** 2025-01-26

**Authors:** Tomáš Toporcer, Štefan Lukačín, Andrea Kraus, Marián Homola, Anton Bereš, Michal Trebišovský, Denis Radótzy, Vilém Rohn, Adrián Kolesár

**Affiliations:** 1 Department of Heart Surgery, East Slovak Institute for Cardiovascular Diseases and Medical Faculty of Pavol Jozef Šafárik University, 04001 Košice, Slovakia; topyto@gmail.com (T.T.); mhomola@vusch.sk (M.H.); aberes@vusch.sk (A.B.); mtrebisovsky@vusch.sk (M.T.); dradotzy@vusch.sk (D.R.); akolesar@vusch.sk (A.K.); 2 Institute of Mathematics and Statistics, Faculty of Science, Masaryk University, 602 00 Brno, Czech Republic; andrea.kraus@mail.muni.cz; 3 II. Surgery Department–Cardiovascular Surgery, General University Hospital and First Faculty of Medicine, Charles University, 128 08 Prague, Czech Republic; vilem.rohn@seznam.cz

**Keywords:** aortic valve replacement, aortic valve stenosis, mechanical prosthesis, bioprosthesis

## Abstract

Background: Aortic valve replacement (AVR) is the definitive therapy for patients with severe aortic valve stenosis (AoS). The aim of this work is to compare the effect of a mechanical prosthesis (MP) and a bioprosthesis (BP) on the survival of patients aged 50–65 years after AVR. Methods: The retrospective analysis included 276 patients aged 50 to 65 years who had undergone isolated AVR for AoS; 161 patients were implanted with an MP and 115 with a BP. Patient survival, adjusted for age, gender and risk parameters affecting survival, was assessed. A subgroup analysis was performed on the 208 patients with a modern valve (prosthesis models that are no longer used in clinical practice were removed from the sample). Results: After adjusting for risk factors for overall survival as well as for age and sex, the implantation of an MP did not have a significant effect on overall survival in comparison to a BP, at a median follow-up of 10.3 years (*p* = 0.477). The size of the MP had no significant effect on overall survival either (HR: 1.29; 95%CI: 0.16–10.21; *p* = 0.812). However, the indexed effective orifice area of the BP had a positive effect on overall survival (HR: 0.09; 95%CI: 0.01–0.78; *p* = 0.029). Conclusions: The estimated survival of patients aged between 50 and 65 years after implantation of a BP with a sufficiently large indexed effective orifice area may exceed that of patients with an MP.

## 1. Introduction

Aortic valve stenosis (AoS) is the most common valve disorder in developed countries. Aortic valve replacement (AVR) is the definitive therapy for patients with severe AoS and for patients with aortic valve regurgitation who are not candidates for aortic valve repair [1]. Worldwide, some 280,000 patients undergo this surgery each year [2]. The choice between a mechanical prosthesis (MP) and a bioprosthesis (BP) in non-elderly patients is a long-standing topic. The guidelines of both the European Society of Cardiology (for patients aged 60–65) and the American College of Cardiology (for patients aged 50–65) make no recommendation for specific prostheses for these patients and leave the choice to the surgeon based on the comorbidities and expected survival of the individual patient [3,4].

Both MPs and BPs present advantages and disadvantages that are supported by the literature and are relevant to patient decision-making. Recent studies indicate a shift away from MPs, with at a least two-fold increase reported in the use of BPs among non-elderly patients between 2000 and 2020 [1,5,6,7,8,9]. Almost all published studies identify MPs as a risk factor for major bleeding due to the requirement for anticoagulation with coumarins [5,10,11,12,13]. However, long-term assessments of stroke risk show ambiguous results [2,14,15]. Several studies have demonstrated comparable stroke risks following the implantation of both MP and BP for aortic valve replacement [10,13]. On the other hand, the use of a BP leads to a higher risk of reoperation, which is also confirmed by a wide range of works [2,5,10,13], though this disadvantage may not be observed in studies with insufficient follow-up duration or small patient populations [14,16]. Lastly, the literature suggests that the risk of prosthetic infectious endocarditis is similar for both types of prostheses [5,17].

Based on this balance between the risk of bleeding and reoperation, the effect of the choice of prosthesis on overall survival appears to be a crucial factor. While older works consistently reported better patient survival after MP implantation, the latest published works no longer unequivocally support this advantage of MPs [5,6,8,10,12]. This temporal direction is not clear-cut; there was even a work published in 2024 that presented the positive effect of MP implantation on the survival of patients aged 45–74 years old [13]. Therefore, the aim of this work is to compare the survival of patients aged 50–65 years after MP and BP implantation, taking into account the current use of valve prostheses.

## 2. Materials and Methods

The processing of the retrospective study was approved by the Ethics Committee in compliance with the principles of the Declaration of Helsinki and ICH Guidelines for Good Clinical Practice and applicable regulatory requirements (protocol code A2062024 and date of approval: 4 June 2024). During hospitalization, patients provided written consent to the healthcare received. According to the recommendation of the Ethics Committee, due to the retrospective nature of the study, no further patient consents were required. All patients aged 50 to 65 years who underwent isolated aortic valve replacement surgery (AVR) because of severe isolated aortic stenosis (AoS) between 2005 and 2016 in our department were included. The choice of prosthesis was made in accordance with the current recommendations of the EACTS and the AHA. Individualization of the prosthesis selection, when permitted by the guidelines, was carried out in line with the patient’s choice after presenting the advantages and disadvantages of each valve prosthesis, based on informed consent. Exclusion criteria were limited to the presence of significant (at least grade 3) aortic valve regurgitation, the need for emergency surgery, the need for concomitant surgery, previous cardiac surgery or infective endocarditis. Patient survival was determined using the database of the health insurance company.

The following data were evaluated: life (at the end of follow-up) [alive/dead]; follow-up time [years]; sex [M/F]; age (at the date of surgery) [years]; prosthesis type [BP/MP]; prosthesis model; indexed effective orifice area of prosthesis (iEOA); ischemic heart disease history (documented stenosis of the coronary artery which was not indicated for revascularization and/or history of a coronary artery intervention) (IHD) [yes/no]; implanted pacemaker before surgery [yes/no]; atrial fibrillation before surgery (AF) [yes/no]; left atrium diameter (LA); left ventricle ejection fraction (LVEF); indexed aortic valve area (iAVA); history of diabetes mellitus (DM) [yes/no]; liver disease (cirrhosis or hepatopathy in anamnesis) (LD) [yes/no]; stroke history [yes/no]; and chronic obstructive pulmonary disease (COPD) [yes/no]. Since some prosthesis models were used on only one to three patients, these patients were excluded from the analyses. In addition, two patients with an unknown prosthesis model were also excluded. Finally, 276 patients aged 50–65 years with five models of MP (Advantage (Medtronic, Minneapolis, MN, USA), ATS (ATS Medical, Inc., Minneapolis, MN, USA), Bicarbon Slimline (Sorin Group /LivaNova PLC, Arvada, CO, USA), On-X (On-X Life Technologies, Inc., Austin, TX, USA) and Regent (St. Jude Medical/Abbott Laboratories, Saint Paul, MN, USA) and five models of BP (Magna Ease (Edwards Lifesciences Corporation, Irvine, CA, USA), Mitroflow (Sorin Group/LivaNova PLC, Arvada, CO, USA), Mosaic (Medtronic, Minneapolis, MN, USA), Soprano (Medtronic, Minneapolis, MN, USA) and Trifecta (Abbott Laboratories/St. Jude Medical, Abbott Park, IL, USA) were included in the analysis.

In a preliminary analysis, the two groups of patients (MP and BP) were compared using Kaplan–Meier survival curves and the log-rank test. Subsequently, a Cox proportional hazards model was used to identify risk factors for the overall survival of patients after surgery. The model was then used to evaluate the effect of prosthesis type (MP vs. BP) on overall survival, adjusted for age, sex and the risk factors for overall survival. Finally, the effects of the iEOA of MP and the iEOA of BP, adjusted for the same characteristics, as above, were evaluated.

In the subsequent subgroup analysis, only patients with a modern prosthesis model that was still currently being implanted as of the date of the manuscript preparation were included. Patients with the four prosthesis models (Advantage, Mitroflow, Soprano and Trifecta) that were not in use in 2024 were excluded from the group. Finally, 146 patients with an MP and 62 patients with a BP were included in the modern model group (*n* = 208). For the statistical evaluation, the same statistical procedures were chosen as for the evaluation of the complete group. Statistical evaluation was performed in the R program, version R 4.4.2, at the level of statistical significance *p* < 0.05 [18].

## 3. Results

Table 1 contains the summary statistics for the continuous variables: number of patients with a non-missing value of the variable (n), minimum (Min), first quartile (q1), median ($\tilde{x}$), third quartile (q3), maximum (Max) and inter-quartile range (IQR). The characteristics are given for all patients together and also separately for the patients with each type of prosthesis. In addition, the *p* values of the Mann–Whitney test for the difference between the characteristics of the patients in the two groups (MP vs. BP) are provided. Furthermore, Table 1 also contains summary statistics for the nominal variables: the number and percentage of patients with a given characteristic. The characteristics are given for all patients together and also separately for the patients with each type of prosthesis. In addition, the *p* values of the Fisher exact test for the difference between the characteristics of the patients in the two groups (MP vs. BP) are provided. The patients in both groups were similar, excluding the follow-up duration, age, IHD and iEOA.

**Figure 1:**
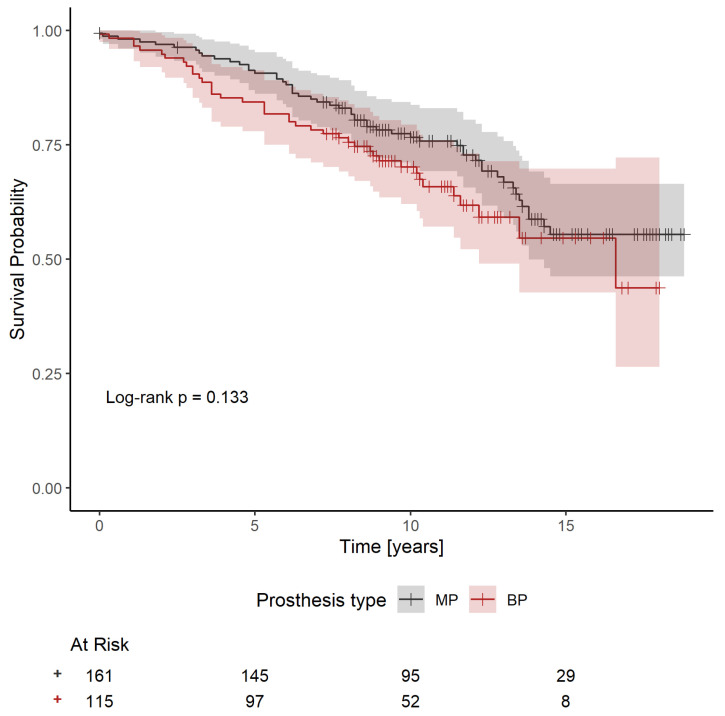
Kaplan–Meier estimates of the survival after surgery for patients with an MP and patients with a BP (*p* = 0.133) (MP—mechanical prosthesis; BP—bioprosthesis).

**Figure 2:**
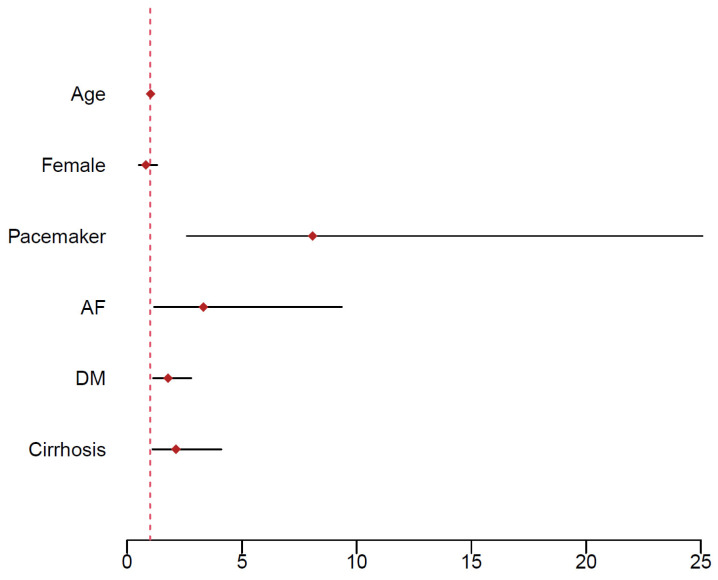
Graphical representation of the results of a Cox proportional hazard model (Age: (HR: 1.022; 95%CI: 0.967–1.080; *p* = 0.439); Female: (HR: 0.814; 95%CI: 0.507–1.307; *p* = 0.394); pacemaker: (HR: 8.080; 95%CI: 2.604–25.072; *p* < 0.001); AF: (HR: 3.323; 95%CI: 1.180–9.360; *p* = 0.023); DM: (HR: 1.782; 95%CI: 1.135–2.799; *p* = 0.012); LD: (HR: 2.127; 95%CI: 1.102–4.107; *p* = 0.025)) (AF—atrial fibrillation; DM—diabetes mellitus).

**Figure 3:**
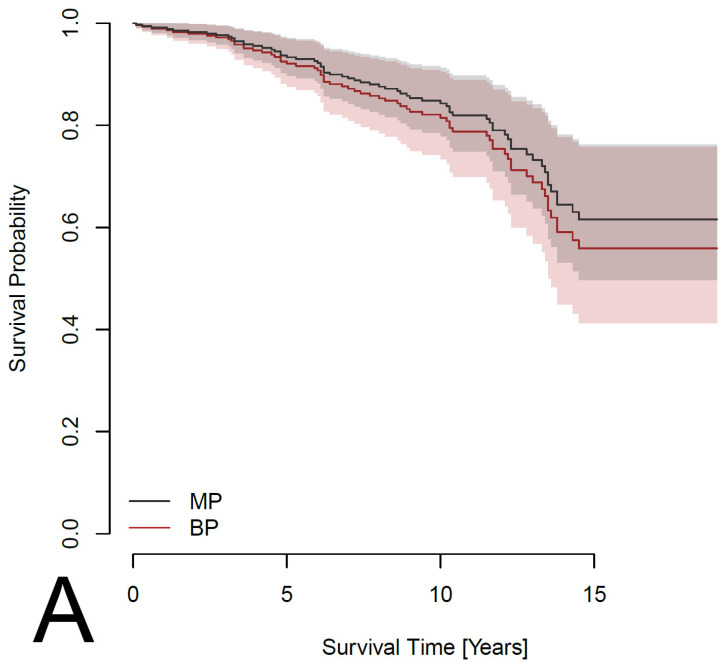

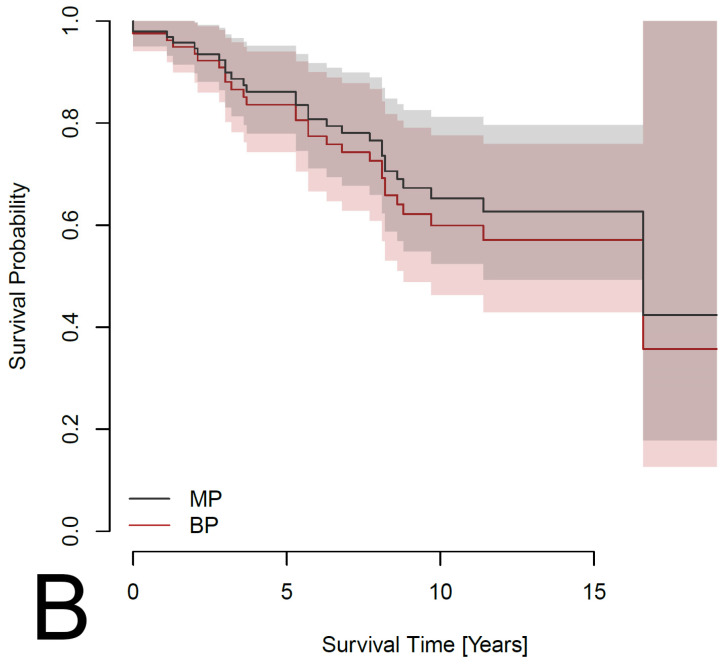
Estimated survival curves from a Cox proportional hazards model comparing patients with the two types of prosthesis (MP—mechanical prosthesis; BP—bioprosthesis). The models are stratified for IHD (ischemic heart disease) and the effect is adjusted for age, sex, pacemaker, AF, DM and LD. The curves are shown separately for patients without (**A**) and with (**B**) an IHD; all curves are computed for patients aged 60 years with no pacemaker, AF, DM or LD.

**Figure 4:**
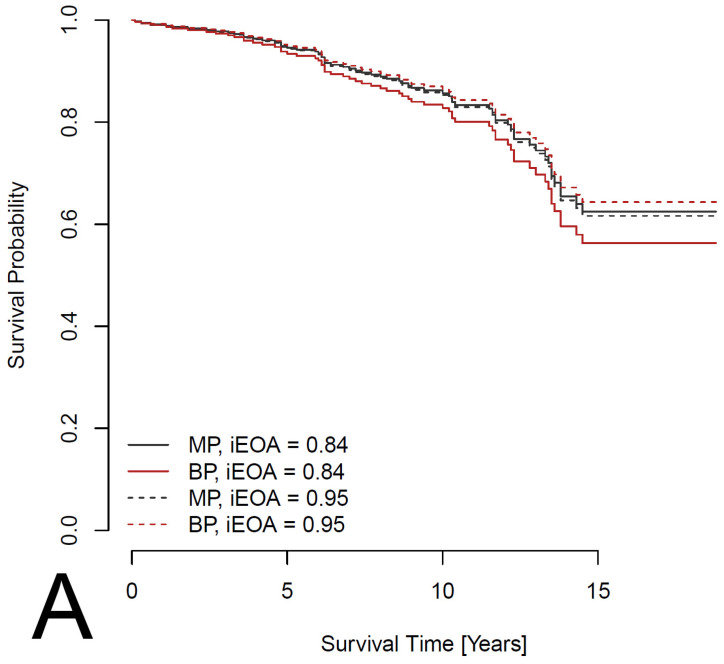

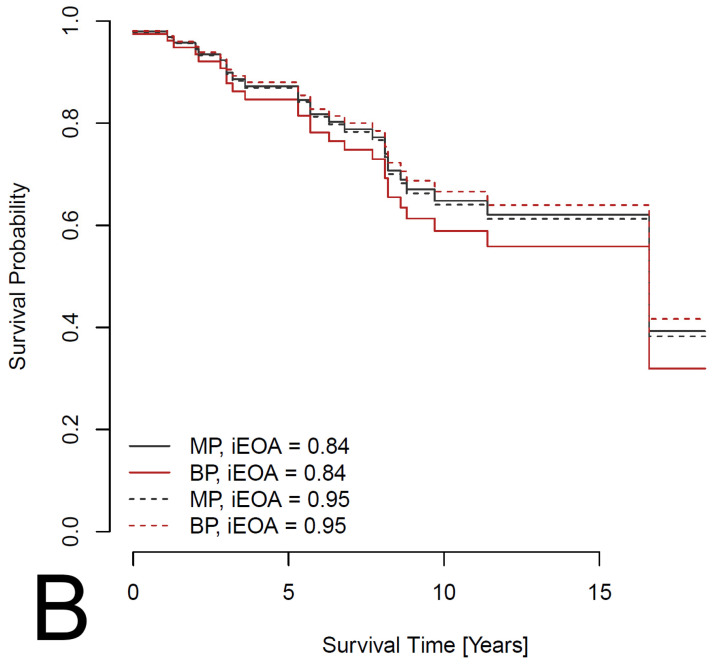
Estimated survival curves from a Cox proportional hazards model comparing patients with the two types of prosthesis (MP—mechanical prosthesis; BP—bioprosthesis) and different values of the iEOA (indexed effective orifice area). The model is stratified for IHD (ischemic heart disease), and the effect is adjusted for age, sex, pacemaker, AF, DM and LD. The curves are shown separately for patients without (**A**) and with (**B**) an IHD; all curves are computed for male patients aged 60 years with no pacemaker, AF, DM or LD.

Preliminary analysis using Kaplan–Meier survival curves and the log-rank test suggested a non-significantly better survival rate (*p* = 0.133) for patients with an MP (Figure 1). However, summary statistics (Table 1) showed that patients with an MP are younger on average and less likely to have a history of IHD. To adjust the effect of MP versus BP for these and other potential confounders, a Cox proportional hazards model was considered, in which survival depends on age, sex, comorbidities (IHD, pacemaker, AF, DM, LD, stroke and COPD) and parameters of the heart (LA, LVEF and iAVA). Out of these, pacemaker (HR: 8.080; 95%CI: 2.604–25.072; *p* < 0.001), AF (HR: 3.323; 95%CI: 1.180–9.360; *p* = 0.023), DM (HR: 1.782; 95%CI: 1.135–2.799; *p* = 0.012) and LD (HR: 2.127; 95%CI: 1.102–4.107; *p* = 0.025) had a significant effect on survival (Figure 2). In addition, the effect of IHD was possibly non-proportional. Subsequently, a Cox proportional hazards model stratified for IHD was used to evaluate the effect of prosthesis type adjusted for age, sex, pacemaker, AF, DM and LD. The effect was found to be non-significant, with a *p*-value even larger than in the preliminary analysis (HR: 1.199; 95%CI: 0.727–1.978; *p* = 0.477). Survival curves fitted by the model comparing patients with the two types of prosthesis are shown in Figure 3. Separate curves were drawn for males and females and for patients with and without an IHD; all curves correspond to patients aged 60 years (the median age of the patients in the database) with no comorbidities, except, potentially, an IHD. Finally, the effects of the iEOA of MP and the iEOA of BP, adjusted for the same factors as above, were evaluated. The size of the iEOA had little effect on the survival of patients with MP (HR: 1.286; 95%CI: 0.162–10.205, *p* = 0.812), while a larger iEOA was associated with the prolonged survival of patients with BP (HR: 0.090; 95%CI: 0.010–0.783; *p* = 0.029). Survival curves fitted by the model comparing patients with the two types of prosthesis and different iEOA sizes are shown in Figure 4. Separate curves are shown for males and females and for patients with and without an IHD; all curves correspond to patients aged 60 years with no comorbidities, except, potentially, an IHD. Two iEOA sizes are shown in the figures: 0.84 (median iEOA for patients with a BP) and 0.95 (median iEOA for patients with an MP). Estimated survival is shortest for patients with a BP with an iEOA of 0.84 and longest for patients with a BP with an iEOA of 0.95, while the estimated survival of patients with an MP is in the middle and does not differ much between the two iEOA sizes.

**Figure 5:**
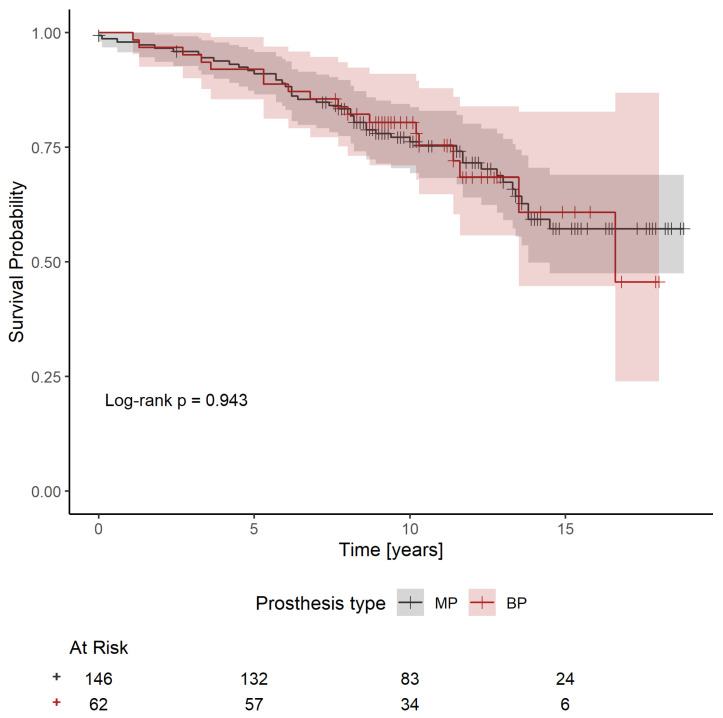
Kaplan–Meier estimates of survival after surgery for patients with a modern model of an MP and patients with a modern model of a BP (*p* = 0.943) (BP—bioprosthesis; MP—mechanical prosthesis).

**Figure 6:**
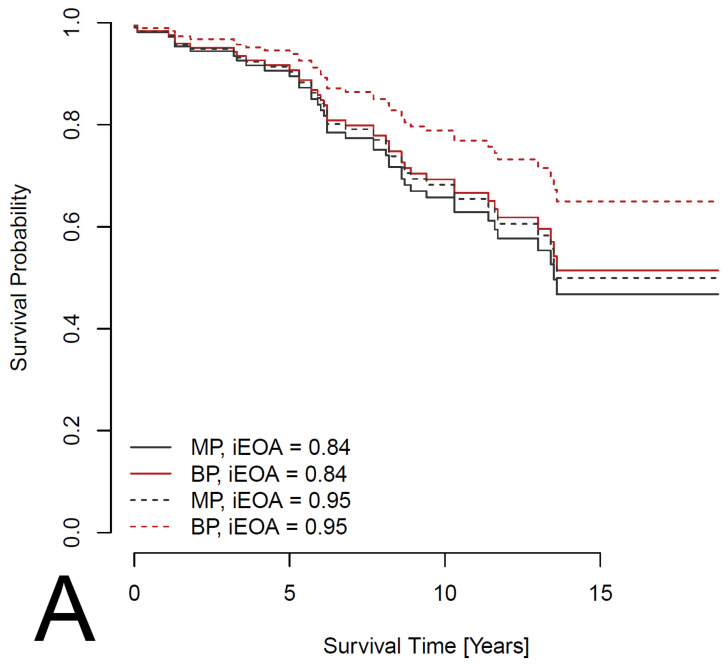

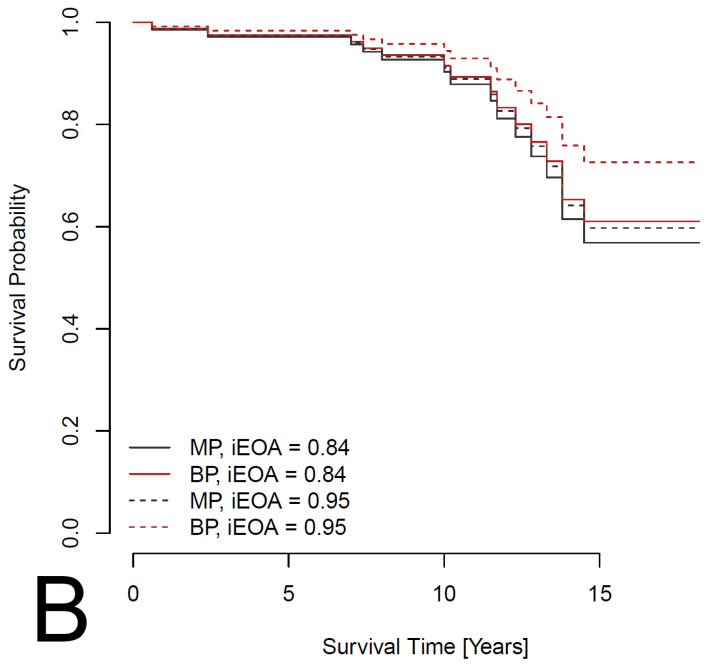
Estimated survival curves from a Cox proportional hazards model comparing patients with the two types of prosthesis (MP—mechanical prosthesis; BP—bioprosthesis) and different values of the iEOA (indexed effective orifice area). The model is stratified for sex, and the effect is adjusted for age, pacemaker and iAVA. The curves are shown separately for male (**A**) and female (**B**) patients; all curves are computed for patients aged 60 with an iAVA value of 0.33 and no pacemaker.

In the subgroup of patients with a modern prosthesis, preliminary analysis based on Kaplan–Meier survival curves and a log-rank test suggested no difference between patients with an MP and those with a BP (*p* = 0.943) (Figure 5). The Cox proportional hazards model only identified having a pacemaker as a significant predictor of overall survival (HR: 8.242; 95%CI: 1.823–37.262; *p* = 0.006), while the effect of indexed AVA was borderline (HR: 0.092; 95%CI: 0.008–1.117; *p* = 0.061), and the effect of sex possibly non-proportional. Using a Cox proportional hazards model stratified for sex, the effect of the type of prosthesis adjusted for pacemaker and iAVA was evaluated as non-significant (HR for a BP: 0.858; 95%CI: 0.471–1.561; *p* = 0.615). Finally, an evaluation of the effect of the iEOA was performed. It is estimated that a larger iEOA value for modern prosthesis models is beneficial both for patients with an MP (HR: 0.436; 95%CI: 0.046–4.115; *p* = 0.468) and, more substantially, for patients with a BP (HR: 0.019; 95%CI: 0.000–2.014; *p* = 0.096). However, the results are not statistically significant. The survival curves fitted by the model are shown in Figure 6.

## 4. Discussion

Two reputable meta-analyses focusing on the effect of prostheses on the survival of non-elderly patients after aortic valve replacement have been published in recent years [10,12]. A study by Diaz et al., involving 4686 patients with propensity score matching, demonstrated statistically significant better survival rates after MP implantation at 5, 10, and 15 years of follow-up. Survival rates were 89.5% for MP and 88.1% for BP at 5 years, 76.8% for MP and 74.1% for BP at 10 years, and 61.6% for MP and 58% for BP at 15 years of follow-up [10]. A meta-analysis by Tasoudis et al. includes 11,169 patients, and the authors concluded that patients with an MP have a decreased hazard for mortality (HR: 0.76; 95%CI: 0.70–0.83; *p* < 0.0001) compared to those with a BP in the age group of 50–70 years old [12]. However, it should not be overlooked that these two meta-analyses include research work that included patients who underwent surgery between 1995–2014 and 1970–2020, and no correction for prostheses still currently in use was performed. The results of original articles focused on survival after MP and BP implantation in patients aged 50–65 published in recent years are more ambiguous. Several of these papers include patients who had surgery after the year 2000 [2,5,6,8,14]. The cohort of patients operated on in the 21st century is also associated with the use of newer BP models. Spanish authors reported no difference in survival in patients who had surgery in 2000–2018 and 2000–2015, and both studies included a follow-up of more than 15 years [5,6]. A work from the United Kingdom (UK), focused on patients in the age range of 50–69 years, did not record any difference in survival during a follow-up of 15 years after MP or BP implantation [8]. Data from the German Aortic Valve Registry (GARY) include a follow-up of only 5 years and did not identify any difference in survival after MP or BP implantation [14]. On the other hand, the AUTHEARTVISIT study (n = 13,993) identified MP replacement as a prognostic factor of survival for patients aged 50–65 years (HR = 1.866; 95%CI 1.448–2.404; *p* < 0.001). The study includes patients who had surgery between 2010 and 2018 [2]. A study by Sun et al. includes patients who underwent surgery from 1989–2019 and also shows better survival after MP implantation [13].

When evaluating these works, we can note a shift from the unequivocal benefits of MP when evaluating the survival of patients undergoing aortic valve surgery in the 20th century to the ambiguous results of MP and BP in patients undergoing surgery after 2000. Patient follow-up is also an important factor in these studies because, according to several studies, survival after the implantation of MP and BP at 5 years follow-up is comparable. Differences between valve replacements are often only identifiable after a 10–15 year horizon [19]. However, none of the mentioned studies examined, in correlation with our results, identify BP as a prognostic factor for survival in patients aged 50–65 years. It can be anticipated that future works, including those involving current models of MP and BP, will show comparable survival of patients aged 50–65 years. An indisputable significant parameter affecting postoperative complications is the ability of patients to adhere to optimal international normalized ratio (INR) values after MP implantation [11]. With comparable results, MP and BP survival, and for a patient aged 50–65 years in whom we assume non-optimal adherence to the optimal INR, BP implantation appears to be safer. The results of this work present the survival of patients who underwent surgery at the beginning of the 21st century. The data thus correlate with more recent studies and at first contradict the conclusions of the often cited meta-analyses involving patients operated on at the end of the 20th century. The initial evaluation of the raw data indicates non-significantly better patient survival after MP implantation (*p* = 0.133). When the effects of parameters that may influence patient survival are taken into account, the effect of valve-type selection was visibly attenuated (*p* = 0.477). Therefore, even with the presented data, we perceive more frequent use of a BP in patients with expected and actual shorter survival. This can greatly affect the validity of studies of unbalanced groups. Fewer than 20% of surgeons in their 50s and 60s would choose an MP for aortic valve replacement for themselves. However, up to 50% of surgeons would recommend an MP implantation to a patient aged 55 years old [20]. The shift towards BP is caused by the increased durability of new BP models, the decrease in morbidity for reoperations, and the possibilities of the valve-in-valve (ViV) method [19]. However, these assumptions are not confirmed, and according to the latest studies, ViV represents only 18.3% of the cases of BP failure [1]. Despite their trust in BP, surgeons follow guidelines and the results of long-term follow-up studies when making recommendations to patients.

The indexed aortic valve area and patient prosthesis mismatch effect are parameters very often debated in the literature. Several authors focused on the effect of a very low iEOA on survival. A paper by Serbian authors focusing on the presence of a patient–prothesis mismatch (PPT) after isolated aortic valve surgery included 595 patients and documented a higher incidence of mild (0.65 cm^2^/m^2^ < iEOA < 0.85 cm^2^/m^2^) and severe (iEOA < 0.65 cm^2^/m^2^) PPT after BP use (69.8% vs. 3.7%) (*p* < 0.001). Moreover, the presence of PPT affected survival at 31.8 months follow-up after biological valve implantation, but not after mechanical valve implantation [21]. On the other hand, data focused on the impact of valve size on survival is lacking. According to our results, the iEOA and size of an implanted valve have a minimal impact on survival if MPs are used. On the other hand, the size of the BP has a significantly positive effect on survival. More importantly, an oversized BP may lead to better survival for a patient aged 50–65 years compared to an MP. Looking at the descriptive statistics of the groups, one can see a statistically significantly greater iEOA in MPs compared to BPs. Accepting the conclusion that a smaller iEOA disadvantages BP, this, too, may in the final consequences negatively affect the erroneous assessment of survival after BP implantation in unbalanced data.

## 5. Limitations of the Study

The main limitation of the study is its retrospective nature and the evaluation of data from only a single center. As a result, the findings may be influenced by the socioeconomic characteristics of the population in the region, as well as the selection of patients for the implantation of different types of valve prosthesis. Statistical analysis, particularly the Cox proportional hazards model, can be used to estimate the effects of prosthesis type and size adjusted for measured imbalances among the patients at baseline. However, more precise and generalizable data can only be obtained from a multicenter randomized prospective study. The study results are also intended for use in future meta-analyses, which can significantly eliminate the adverse impact of the aforementioned study limitations.

## 6. Conclusions

The implantation of an MP in comparison with a BP in an unbalanced group of patients aged 50–65 years represents a slightly better, statistically insignificant (*p* = 0.133) positive effect on their survival at a median follow-up of 10.3 years. After adjusting the effect for survival risk factors, age and sex, an even smaller difference was observed between the groups (MP vs. BP) (*p* = 0.477). The size of an MP did not have a significant effect on survival, while the iEOA of a BP had a positive effect on the overall survival of patients aged 50–65 years after aortic valve replacement (HR: 0.090; 95%CI: 0.010–0.783; *p* = 0.029).

After excluding prosthesis models whose use has been discontinued, the estimated survival curves suggest that a BP with a larger iEOA may outperform not only BPs with smaller iEOAs but also MPs of comparable sizes. However, additional studies are needed to clearly confirm this hypothesis.

## Figures and Tables

**Table 1:** Descriptive statistics for continuous and nominal variables: %all—percentage of all patients; %BP—percentage of patients with bioprosthesis; %MP—percentage of patients with mechanical prosthesis; AF—atrial fibrillation; COPB—chronic obstructive pulmonary disease; DM—diabetes mellitus; iAVA—indexed aortic valve area; iEOA—indexed effective orifice area; IHD—ischemic heart disease; IQR—inter-quartile range (first quartile–third quartile); LA diam—left atrium diameter; LD—liver disease; LVEF—left ventricle ejection fraction; Max—maximum; Med—median; Min—minimum; n—number of patients with a non-missing value of the variable; nall—number of all patients; nBP—number of patients with a bioprosthesis; nMP—number of patients with a mechanical prosthesis.

Continuous Variable	Levels	n	Min	Med	Max	IQR	#NA
Follow-up	MP	161	0.0	10.7	18.8	8.2–13.9	0
	BP	115	0.0	9.5	18	7.7–11.6	0
*p* = 0.0025	all	276	0.0	10.3	18.8	8.0–13	0
Age	MP	161	49.8	58.3	64.9	55–60.7	0
	BP	115	54.6	62.6	65	61.1–64.2	0
*p* < 0.0001	all	276	49.8	60.5	65	57.2–62.9	0
iEOA	MP	153	0.6	1	1.4	0.9–1.1	8
	BP	112	0.5	0.8	1.3	0.7–0.9	3
*p* < 0.0001	all	265	0.5	0.9	1.4	0.8–1	11
LA diam	MP	122	27	42	62	38–45.8	39
	BP	92	26	44	58	39.8–48	23
*p* = 0.07	all	214	26	43	62	38.2–47	62
LVEF	MP	143	15	55	70	50–60	18
	BP	110	20	55	70	50–60	5
*p* = 0.84	all	253	15	55	70	50–60	23
iAVA	MP	136	0.1	0.3	0.7	0.3–0.4	25
	BP	106	0.1	0.3	0.7	0.2–0.4	9
*p* = 0.69	all	242	0.1	0.3	0.7	0.3–0.4	3
Nominal Variable	Levels	nMP	%MP	nBP	%BP	nall	%all
Sex	M	103	64	63	54.8	166	60.1
	F	58	36	52	45.2	110	39.9
*p* = 0.14	all	161	100	115	100	276	100
IHD	no	132	82	79	68.7	211	76.5
	yes	29	18	36	21.3	65	23.6
*p* = 0.01	all	161	100	115	100	276	100
Pacemaker	no	159	98.8	12	97.4	271	98.2
	yes	2	1.2	3	2.6	5	1.8
*p* = 0.65	all	161	100	115	100	276	100
AF	no	258	98.1	112	97.4	270	97.8
	yes	3	1.9	3	2.6	6	2.2
*p* = 0.7	all	161	100	115	100	276	100
DM	no	131	81.4	89	77.4	220	79.7
	yes	30	18.6	26	22.6	56	20.3
*p* = 0.45	all	161	100	115	100	276	100
LD	no	148	91.9	105	91.3	252	91.7
	yes	13	8.1	10	8.7	23	8.3
*p* = 1	all	161	100	115	100	276	100
Stroke	no	153	95	109	94.8	262	94.9
	yes	8	5	6	5.2	14	5.1
*p* = 1	all	161	100	115	100	276	100
COPD	no	145	90.1	103	89.6	248	89.9
	yes	16	9.9	12	10.4	28	10.1
*p* = 1	all	161	100	115	100	276	100

## Data Availability

All important data are contained in the text of the manuscript. Raw data are available to the journal editor if requested.

## Abbreviations

AF	atrial fibrillation
AoS	aortic valve stenosis
AVR	aortic valve replacement surgery
BP	bioprosthesis
COPD	chronic obstructive pulmonary disease
DM	diabetes mellitus
iAVA	indexed aortic valve area
iEOA	indexed effective orifice area
IHD	ischemic heart disease history
INR	international normalized ratio
LA	left atrium
LD	liver disease
LVEF	left ventricle ejection fraction
MP	mechanical prosthesis
ViV	valve-in-valve
